# Diabetic foot ulcer wound fluid: the effects of pH on DFU bacteria and infection

**DOI:** 10.1186/1757-1146-8-S1-A8

**Published:** 2015-04-20

**Authors:** Carla McArdle, Katie Lagan, Sarah Spence, David McDowell

**Affiliations:** 1 Centre for Health and Rehabilitation Technologies (CHaRT), School of Health Sciences, University of Ulster, Newtonabbey, UK; 2 School of Health Sciences, University of Ulster, Newtonabbey, UK

## Background

Foot ulceration is one of the most significant complications of diabetes, and will affect 15-20% of people with diabetes at some point in their lives. Such ulcers frequently become infected with very serious sequelae which can often lead to amputation making diabetes is the most common cause of lower extremity amputations. Infections cause increased morbidity (and/or mortality) which means that they represent significant clinical events, requiring immediate attention in relation to local and systemic complications thus requiring well-coordinated management. Unfortunately diabetic foot infections (DFI) frequently fail to display overt signs and symptoms of infection including purulence, erythema, pain, tenderness, warmth and induration. This makes it difficult for clinicians to detect infection, and to make timely interventions to limit the highly undesirable consequences of DFIs. Alternative means of rapidly diagnosing infection are urgently required.

## Aim

To determine if the presence/absence of microorganisms, and ultimately the presence of infection, are affected by diabetic foot ulcer (DFU) wound fluid pH.

## Methods

DFUs of patients (n=55) were assessed in terms of presence/absence of clinical signs of infection as part of their routine clinical appointment at a High-Risk Foot Clinic. Wound fluid samples were also collected from the DFUs by filter paper absorption and/or pipette aspiration. The pH of samples was determined using a micro-electrode pH meter. Bacteria in the wound fluid were recovered by 24hr incubation in Tryptone Soya Broth, and plating on selective agars which included; MacConkey Agar (Staphylococcus spp, Enterobacter spp), Pseudomonas Agar Base (Pseudomonas spp). Chromocult Agar (E. Coli, Coliforms), Baird Parker Agar (Staphylococcus spp) and Columbia Blood Agar (Streptococcus spp). Organisms identified as Staphylococcus Aureus cultured on Muller Hinton Agar and and MRSA present detected using Oxacillin and Cefoxitin antibiotic disks.

## Results

Sample pH values ranged from 6.2 to 8.5. Recovered bacteria included Pseudomonas, Enterobacter, Staphylococcus and Streptococcus spp. Correlations were observed between DFU fluid pH values, the presence/absence of these species and the presence/absence of clinical signs of infection. This presentation will discuss the potential clinical implications of these findings.

## Conclusions

pH conditions within DFUs influence bacterial presence/absence in these wounds. pH conditions also influence the presence/absence of clinical signs of infection. Timely monitoring of DFU fluid pH could enhance clinicians’ abilities to rapidly detect and more effectively manage DFU infections. An improved understanding of the interactions between DFU pH and bacterial metabolism may identify ways to limit the duration and wider impact of DFU infections.

Governance compliance statement - Ethical approval and research governance has been granted for the clinical study to take place and all research governance procedures are being adhered to during the completion of the study.
